# Transcriptional Analysis of FOXO1, C/EBP-α and PPAR-γ2 Genes and Their Association with Obesity-Related Insulin Resistance

**DOI:** 10.3390/genes10090706

**Published:** 2019-09-12

**Authors:** Hatim Boughanem, Amanda Cabrera-Mulero, Mercedes Millán-Gómez, Lourdes Garrido-Sánchez, Fernando Cardona, Francisco José Tinahones, Inmaculada Moreno-Santos, Manuel Macías-González

**Affiliations:** 1 Biomedical Research Institute of Malaga (IBIMA), Faculty of Science, University of Malaga, 29010 Málaga, Spain; h.b.boughanem@gmail.com; 2 Department of Endocrinology and Nutrition, Virgen de la Victoria University Hospital, University of Malaga (IBIMA), 29010 Málaga, Spain; amanda.c.mulero@hotmail.com (A.C.-M.); lourgarrido@gmail.com (L.G.-S.); fjtinahones@hotmail.com (F.J.T.); 3 CIBEROBN (CIBER in Physiopathology of Obesity and Nutrition CB06/03/0018), “Instituto de Salud Carlos III”, 28029 Madrid, Spain; 4 CIBERCV (CIBER in cardiovascular diseases), “Instituto de Salud Carlos III”, 28029 Madrid, Spain; mercedesmillang@gmail.com; 5 Unidad de Gestión Clínica Área del Corazón, Virgen de la Victoria University Hospital, University of Malaga (IBIMA), 29010 Málaga, Spain

**Keywords:** C/EBP-α, PPAR-γ2, FOXO1, IGFBP-2, obesity, insulin resistance

## Abstract

Background: Obesity is associated with several comorbid disorders, ranging from cardiovascular diseases to insulin resistance. In this context, visceral adipose tissue (VAT) seems to have a close connection with insulin resistance. In our study, we hypothesized that the expression profile of key adipogenic genes, such as proliferator-activated receptor γ type 2 (PPAR-γ2), CCAAT/enhancer-binding protein type α (C/EBP-α), and forkhead box protein class O type 1 (FOXO1) in VAT should shed light on their association with obesity-related insulin resistance. Methods: To test this idea, we studied the expression profile of C/EBP-α, FOXO1 and PPAR-γ2 in VAT from non-obese individuals, and low insulin (LIR-MO) and high insulin morbidly obese (HIR-MO) subjects, through a combination of RT-qPCR, co-immunoprecipitation, ELISA, Western blot analysis and EMSA assays. Results: Our results show that C/EBP-α and PPAR-γ2 were down-expressed in HIR-MO individuals, while FOXO1 was overexpressed. In addition, the PPAR-γ2–RXR-α heterodimer showed weak activity and bound weakly to the putative IGFBP-2–PPRE promoter sequence in VAT from HIR-MO subjects when compared with LIR-MO individuals. Conclusions: These results show that PPAR-γ2, C/EBP-α, FOXO1 and IGFBP-2 have a close relationship with insulin resistance in VAT of morbidly obese individuals.

## 1. Introduction

Obesity is a serious public health problem that has become a global epidemic, and its incidence is dramatically increasing [1]. Overall excess of fat has long been linked to many different underlying non-communicable diseases, including cardiovascular diseases, certain cancers and metabolic disorders, such as type 2 diabetes and insulin resistance [2]. The adverse health consequences of obesity and insulin resistance are well-documented, particularly with respect type 2 diabetes mellitus, and it was recently linked to neurological disorders, including Alzheimer’s disease [3,4]. In this sense, both adipocytes differentiation and expandability of adipose tissue are key players in the physiopathology of obesity-related insulin resistance [5]. During adipogenesis, several transcription factors activate/repress the expression of crucial adipogenic genes in the visceral adipose tissue (VAT) to induce insulin sensitivity [6,7].

Peroxisome proliferator-activated receptor γ type 2 (PPAR-γ2) and CCAAT/enhancer-binding protein type α (C/EBP-α) are two transcription factors that are considered the masters of adipogenesis [8]. PPAR-γ2 is highly expressed in adipose tissue, although it has been reported that PPAR-γ2 in mice is ectopically induced in important metabolic tissue, such as the liver and skeletal muscle, in response to overnutrition and genetic obesity [9]. The majority of promoters/enhancers of principal adipogenic genes have PPAR-γ DNA binding sites [10]. Therefore, the activation of PPAR-γ by ligands has antidiabetic actions and improve insulin sensitivity, although the mechanism is not fully understood [11]. Alternatively, C/EBP-α is expressed during adipocytes differentiation in culture cells at later stages, and it stays active in mature adipocytes [12]. Ectopic expression of C/EBP-α in NIH-3T3 fibroblastic cell line are sufficient to induce them to differentiate into adipocytic cells. Although cells deficient in C/EBP-α were able to initiate adipocyte differentiation, these cells were insulin resistant [8,13].

The forkhead box protein class O type 1 (FOXO1) also participates in adipogenesis, by preventing adipose tissue differentiation through the inhibition of PPAR-γ transcription. The mechanism of PPAR-γ inhibition occurs by physically interacting with PPAR-γ and binding to its promoter site [14,15,16,17]. Additionally, FOXO1 directly interacts with C/EBP-α, regulating its function and link the insulin signaling to C/EBP-α [18]. Thus, FOXO1 is one of the crucial metabolic links between PPAR-γ and C/EBP-α, suggesting an interesting role that FOXO1 must play in adipogenesis and insulin resistance.

Insulin growth factor-binding proteins (IGFBPs) have been shown to have an interesting effect to promote adipocyte differentiation [19]. In fact, IGFBP type 2 (IGFBP-2) is predominantly produced by preadipocytes during adipogenesis [20]. Circulating levels of IGFBP-2 are suppressed in obese individuals, an effect which is more linked to visceral adiposity, suggesting a novel role of IGFBP-2 in obesity prevention [21,22,23]. A previous study showed that the incubation of adipocytes with insulin triggered the recruitment of C/EBP-α to the proximal region of the IGFBP-2 promoter, indicating that C/EBP-α could regulate IGFBP-2 expression in an insulin-dependent manner [24]. Nevertheless, no data are available on whether PPAR-γ2 can activate IGFBP-2 by binding on its promoter site.

Considering the complex role of PPAR-γ2, C/EBP-α and FOXO1 in adipogenesis and insulin resistance, we propose that their expression profile in VAT from non-obese individuals and low insulin (LIR-MO) and high insulin morbidly obese (HIR-MO) subjects might shed light on their possible association with obesity-related insulin resistance. In addition, given the anti-obesity and anti-diabetic properties of IGFBP-2, we suggest that IGFBP-2 might interact with PPAR-γ2 to protect against insulin resistance in obese individuals. To test this hypothesis, we set out to undertake a new context to determine the gene expression of PPAR-γ2, C/EBP-α and FOXO1 from morbidly obese individuals with high and low insulin resistance in VAT, to understand their association with obesity and insulin resistance. Further, we determined the PPAR-γ2–RXR-α heterodimer activity in VAT, especially its interaction on the IGFBP-2 promoter to test its association with obesity and insulin resistance.

## 2. Materials and Methods 

### 2.1. Study Designs and Participants

In the present study, we included 38 participants from the “Virgen de la Victoria” University Hospital (Málaga, Spain). Twenty-three were morbidly obese (MO) (body mass index (BMI) > 40 kg/m^2^) subjects and the remaining 15 were non-obese (BMI < 25 kg/m^2^) individuals. The MO group was also classified in two groups according to their insulin resistance state: 11 low insulin resistance MO (LIR-MO) (Homeostasis model assessment of insulin resistance (HOMA-IR) < 5) and 12 high insulin resistance MO (HIR-MO) (HOMA-IR > 8) subjects [7,25,26,27,28,29,30]. The weight of MO and non-obese individuals was stable in the last six months prior to participation in the study. The exclusion criteria were patients who have diabetes mellitus, coronary artery disease, acute or chronic inflammatory diseases, renal and infectious diseases, patients who have received treatment that alters the glucose profile such as metformin and lipid profile as statins, have altered others metabolic parameters, or consumed > 20 g of ethanol per day at the time of inclusion in the present study. Non-obese subjects had undergone laparoscopic surgery for elective cholecystectomy or hiatal-hernia surgery and VAT samples were obtained from mesenteric depot. MO patients had undergone Bariatric surgery procedures and VAT biopsies were obtained from the greater omentum. All tissues were frozen immediately after surgery in liquid nitrogen and stored at −80 °C for RNA and protein isolation. All participants were anonymized and gave their written informed consent. The study was performed in accordance with the “Declaration of Helsinki” and approved by the Ethics Committees of “Virgen de la Victoria” University Hospital (Málaga, Spain, 0311/PI7).

### 2.2. Analytical Methods and Laboratory Measurements

Blood samples from all participants were obtained after an overnight fast and before surgery. Glucose, triglycerides, total cholesterol, high-density lipoprotein cholesterol (HDL) and C-reactive protein (CRP) measurements were performed using a Dimension Autoanalyzer (Dade Behring Inc., Deerfield, IL, USA). The insulin was measured by an immunoradiometric assay (BioSource International, Camarillo, CA, USA). All biochemical parameters were measured in duplicate. Calculated values for low-density lipoprotein cholesterol (LDL) was obtained by the Friedewald formula [31]. The HOMA-IR was determined using the following equation: HOMA-IR = fasting insulin (μIU/mL) × fasting glucose (mmol/L)/22.5 [32].

### 2.3. Total RNA Extraction and qPCR

Total RNA isolation from VAT (~100 mg) was performed using RNeasy Lipid Tissue Mini Kit (Qiagen GmbH, Hilden, Germany), according to the manufacturer’s instructions. To generate first-strand cDNA synthesis, we used 1 μg of total RNA, Moloney Murine Leukemia Virus reverse transcriptase and random hexamers primers (Roche Diagnostic, Rotkreuz, Switzerland), as indicated by the manufacturer. The specific primers for PPAR-γ2, C/EBP-α and FOXO1 were designed using Primer-Blast (NCBI) and were synthesized at Sigma-Aldrich (Sigma Aldrich, Madrid, Spain). qPCR was performed using LightCycler technology as described by the provider (Roche Diagnostic, Rotkreuz, Switzerland), with SYBR Green detection. Gene expression was normalized using β-actin as internal control; β-actin gene was selected as an appropriate reference gene from validation methods described previously and used to determine Δ*C*t values [33,34]. Changes of gene expression were calculated by the 2^−ΔΔ*C*t^ method [35]. The results of the expression were represented as the target gene/β-actin ratio.

### 2.4. Protein Isolation, Specific Detection and Quantification

Cytoplasmic and nuclear extracts from VAT of non-obese, LIR-MO and HIR-MO subjects were prepared using NE-PER nuclear and cytoplasmic extraction reagent kit (Thermo Scientific, Rockford, IL, USA). Total cytoplasmic and nuclear protein concentration were quantified by the Bradford assay (Bio-Rad, Richmond, CA, USA), using bovine serum albumin as a standard. Thirty micrograms of total protein extracts were separated by SDS-PAGE. After that, total proteins were blotted onto Polyvinylidene difluoride (PVDF) membrane, and then incubated with specific antibodies, polyclonal anti-C/EBP-α and anti-FOXO1 (Santa Cruz Biotechnology-Sigma Antibodies, Heidelberg, Germany). PVDF membrane containing blotted protein was incubated with the respective secondary polyclonal anti-IgG antibodies (Santa Cruz Biotechnology-Sigma Antibodies, Heidelberg, Germany). Protein signals were visualized using Supersignal West Pico Western blot detection kit (Thermofisher Scientific Pierce Protein Biology, Waltham, MA, USA) and visualized by electrochemiluminescence detection Auto-Chemi system analysis software Labworks 4.6 (UVP; Bio-Imaging Systems DBA Analytik Jena US). To confirm Western blot analysis of C/EBP-α, we used the ELISA assay to quantify C/EBP-α in VAT. Nuclear extracts were prepared with a commercially available kit (Nuclear Extract Kit, Active Motif, Carlsbad, CA, USA), according to the manufacturer’s guidelines. Nuclear extracts were quantified for C/EBP-α using ELISA-based kits (TransAM™, Active Motif, California, Cat No. 44196), following the manufacturer’s instructions. To specifically detect the heterodimer PPAR-γ2–RXR-α, a ligand-immunoblot analysis was performed, in the presence of rosiglitazone (10 μM), as previously published [26].

### 2.5. PPAR-γ2 Immunohistochemistry

Immunostaining for PPAR-γ2 was performed on 25 μm of adipose tissue sections. Sections were fixed overnight at 4 °C in 4% paraformaldehyde and processed for standard paraffin embedding. The sections were treated with PBS containing 10% methanol and 10% hydrogen peroxide for 30 min at room temperature. After that, the sections were incubated overnight, with the polyclonal anti-PPAR-γ2 antibodies (Santa Cruz Biotechnology-Sigma Antibodies, Madrid, Spain). After washing, sections were incubated at room temperature with biotinylated secondary antibodies, for 90 min for Extravidin-peroxidase. The immune peroxidase activity was developed in 3,3′-diaminobenzidine hydrochloride (Vector Laboratories, Peterborough, UK). Sections were counterstained with Mayer’s Hematoxylin (Sigma Aldrich, Madrid, Spain) and mounted in Entellan (Merck, Darmstadt, Germany).

### 2.6. Electrophoretic Mobility Shift Assay (EMSA) for PPAR-γ2

EMSA were performed using 25–50 mg of nuclear extracts from VAT. For supershift assays, 1 μg of polyclonal anti-PPAR-γ2 antibody (Santa Cruz Biotechnology, Inc., CA, USA), 5 μg of bacterially expressed Glutathione S-transferase steroid receptor co-activator type 1 (GST-SRC1_597-791_) or Glutathione S-transferase (GST) as a negative control, were added to 1 ng of the ^32^P-labeled double-stranded oligonucleotides (50,000 cpm) corresponding to four copies of the human CPT1 gene DR1-type PPRE (core sequence 5′-GTAGGGAAAAGGTCA-3′). The mixture was incubated for 15 min at room temperature. Protein–DNA complexes were resolved by electrophoresis through 8% non-denaturing polyacrylamide gels in 0.5× TBE buffer. To evaluate the effect of PPAR-γ2 on the IGFBP-2 promoter, 5 μg nuclear extracts were tested using consensus oligonucleotide for PPRE. Biotin 3′end-labeled probes (Eurogentec, Liège, Belgium) were prepared by annealing oligonucleotides (oligonucleotides for hIGFBP-2 PPRE (5′-ATACGGGAAAGGTCATGAG-3′)). The reaction was performed for 20 min at room temperature. The reaction mixture was revealed in an electrophoresis gel on a 5–10% native polyacrylamide gel in 0.5× TBE buffer and transferred to a nylon membrane (Thermofisher Scientific Pierce Protein Biology, Waltham, MA, USA). Protein-DNA complex was fixed to the nylon membrane by UV and detected by a nonradioactive nucleic acid detection, using the LightShift chemiluminescent EMSA kit (Thermofisher Scientific Pierce Protein Biology, Waltham, MA, USA). For competition studies, DNA binding reaction mixtures were preincubated with unlabeled double strand DNA oligos of the wild type and mutant response element sequences. For supershift assays, 1 μL of specific anti-PPAR-γ2 antibody (Santa Cruz Biotechnology, Inc., Santa Cruz, CA, USA) was preincubated in the binding reaction for 10 min before the probe was added.

### 2.7. Statistical Analysis

The statistical analysis was performed using SPSS (Version 11.5 for Windows; SPSS, Chicago, IL). Comparison among LIR-MO, HIR-MO and non-obese individuals were made using Student’s test for parametric variables. Kruskal–Wallis test was used to evaluate the difference among LIR-MO, HIR-MO and non-obese subjects for non-parametric variables. Wilcoxon test was used to perform the comparison of the results obtained from different adipose tissue. The results are given as mean ± standard deviation (SD). Values were considered to be statistically significant when the *p* < 0.05.

## 3. Results

### 3.1. General Characteristic Data of the Participants

The anthropometric and biochemical variables of the non-obese, LIR-MO and HIR-MO individuals are summarized in Table 1, and partially previously published by our group [26]. While there were no significant differences in sex, age, total cholesterol, triglycerides, HDL and LDL among study groups, MO individuals showed significantly increased value of weight, waist circumference, BMI, insulin and HOMA-IR when compared to non-obese subjects (*p* < 0.05). Within the MO group, the HIR-MO subjects presented significantly higher levels of glucose, insulin and HOMA-IR in comparison with LIR-MO individuals (*p* < 0.05).

### 3.2. The Expression Profile of C/EBP-α and FOXO1 are Altered in VAT from Morbidly Obese Individuals and Associated with Insulin Resistance

**Figure 1:**
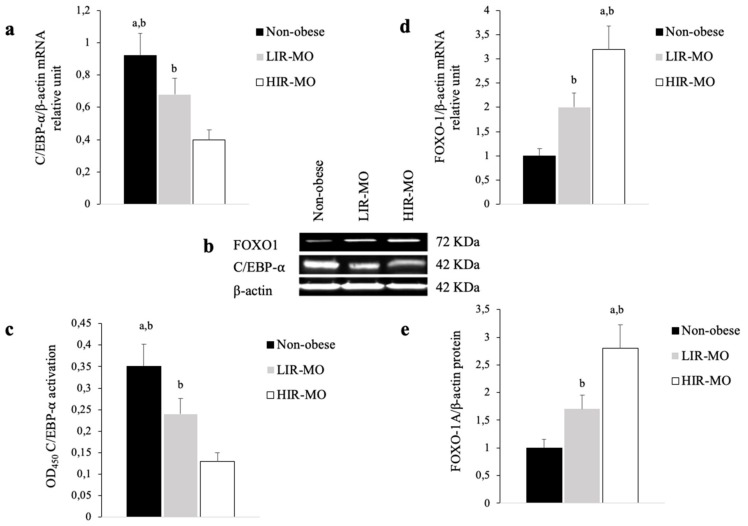
C/EBP-⍺ and FOXO1 gene expression profile in obesity-related insulin resistance. (**a**) Quantitative RT-PCR was used to determine the expression of C/EBP-⍺ mRNA in VAT from non-obese (black bar) (*n* = 15), LIR-MO (grey bar) (*n* = 11) and HIR-MO (white bar) (*n* = 12) individuals. The mRNA expression of C/EBP-⍺ was normalized to β-actin expression. The results are given as the mRNA relative mean expression ± SD. (**b**) Thirty micrograms of total protein extracts from VAT of non-obese (*n* = 15), LIR-MO (*n* = 11) and HIR-MO (*n* = 12) subjects were immunoblotted with anti-C/EBP-⍺, anti-FOXO1 and anti-β-actin antibodies. (**c**) ELISA assay was used to quantify the C/EBP-⍺ protein from nuclear extract from VAT of non-obese (*n* = 15), LIR-MO (*n* = 11) and HIR-MO (*n* = 12) subjects. The results are presented as OD at 450 nm. (**d**) Quantitative mRNA of FOXO1 from VAT from non-obese (*n* = 15), LIR-MO (*n* = 11) and HIR-MO (*n* = 15) subjects. The expression of FOXO1 was normalized using β-actin. (**e**) Total protein extracts from VAT of non-obese (*n* = 15), LIR-MO (*n* = 11) and HIR-MO (*n* = 12) subjects were immunoblotted with anti-FOXO1 and anti-β-actin antibodies. Different letters indicate significant differences between the means of the different groups of subjects (significance of difference: a, *p* < 0.05 controls vs. LIR-MO; b, *p* < 0.05 non-obese vs. HIR-MO; c, *p* < 0.05 LIR-MO vs. HIR-MO), Kruskal–Wallis test followed by Dunn’s test. Abbreviations: C/EBP-⍺, CCAAT/enhancer-binding protein type α; VAT, Visceral adipose tissue; LIR-MO, Low insulin resistance-morbid obese; HIR-MO, High insulin resistance-morbid obese; FOXO1, Forkhead box protein class O type 1; KDa, Kilodalton; OD, Optical density.

A specific gene expression profile of C/EBP-α was found, according to the insulin resistance in morbidly obese individuals (Figure 1a). RT-PCR analysis showed that the expression of C/EBP-α was decreased in morbidly obese subjects (*p* < 0.05). In fact, this expression profile was tightly related to insulin resistance, since HIR-MO individuals had significantly decreased expression of C/EBP-α, in comparison with LIR-MO subjects (*p* < 0.05) (Figure 1a). To confirm these results, we next investigated C/EBP-α protein levels by ELISA and immunoblotting assay. Our results show similar profiles according to RT-PCR assay (Figure 1b,c). In both assays, C/EBP-α was abundantly present in the nuclear extract from non-obese subjects, but only a small amount was detected in LIR-MO and HIR-MO individuals. This amount was significantly decreased in HIR-MO individuals, in comparison with LIR-MO subjects.

We further investigated the gene expression profile of FOXO1 in VAT from non-obese and MO subjects. The finding profile in our study showed that FOXO1 was overexpressed in MO group, for which the mRNA levels of FOXO1 were significantly increased in comparison with non-obese individuals (*p* < 0.05) (Figure 1d). Moreover, within the MO group, FOXO1 expression was significantly higher in VAT from HIR-MO individuals, when compared to LIR-MO individuals (*p* < 0.05). We further examined FOXO1 protein levels in nuclear extracts, to affirm the RT-PCR results. In support of our results, the Western blot assay was similar to mRNA expression FOXO1, showing significantly higher FOXO1 protein levels in MO individuals (*p* < 0.05) and significantly increased FOXO1 protein levels in HIR-MO in comparison with LIR-MO (*p* < 0.05) (Figure 1b,e).

### 3.3. The Expression of PPAR-γ2 is Decreased and Related to Insulin Resistance in VAT from Morbidly Obese Individuals

**Figure 2:**
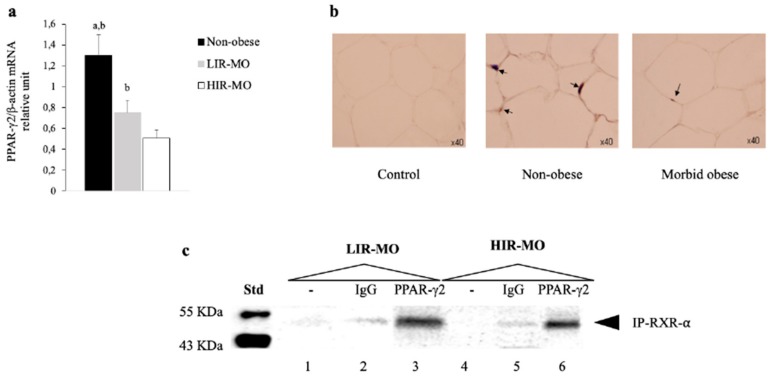
PPAR-γ2 in VAT: A broader view. (**a**) RT-PCR analysis of PPAR-γ2 gene expression in VAT from non-obese (black bar) (*n* = 15), LIR-MO (grey bar) (*n* = 11) and HIR-MO (white bar) (*n* = 12) individuals. Gene expression of PPAR-γ2 was normalized to β-actin expression. The results are given as the mRNA relative mean expression ± SD. (**b**) Immunohistochemical staining for the identification of PPAR-γ2 isoform in adipose tissue section from non-obese (*n* = 15) and morbidly obese individuals (LIR-MO+HIR-MO) (*n* = 23), using specific anti-PPAR-γ2 antibodies. PPAR-γ2 positive staining are showed by arrows. (**c**) PPAR-γ2 coimmunoprecipitates with RXR-⍺ in nuclear extracts from VAT of the LIR-MO (*n* = 11) and HIR-MO (*n* = 12) subjects. One hundred micrograms of nuclear extracts of the VAT expressing PPAR-γ2 were preincubated with rosiglitazone (PPAR-γ2 agonist) (10 µM) for 4 h before being subjected to immunoprecipitation using rat antihuman RXR-⍺ antibodies (Lanes 3 and 6) and purified rat IgG (Lanes 2 and 5) as a control. PPAR-γ2–RXR-⍺ heterodimer recovered by immunoprecipitation were detected by Western immunoblot analysis using rabbit antihuman PPAR-γ2 antibodies. Ten micrograms of the soluble nuclear (Lanes 1 and 4) extracts were also analyzed to evaluate the relative abundance of the protein target. Different letters indicate significant differences between the means of the different groups of subjects (significance of difference: a, *p* < 0.05 controls vs. LIR-MO; b, *p* < 0.05 non-obese vs. HIR-MO; c, *p* < 0.05 LIR-MO vs. HIR-MO), Kruskal–Wallis test followed by Dunn’s test. Abbreviations: VAT, Visceral adipose tissue; RT-PCR, Real time polymerase chain reaction; PPAR-γ2, peroxisome proliferator-activated receptor γ type 2; LIR-MO, Low insulin resistance-morbid obese; HIR-MO, High insulin resistance-morbid obese; KDa, Kilodalton. RXR-⍺, Retinoid X receptor type α.

We first determined the profile of gene expression of PPAR-γ2 in VAT from non-obese and obese morbid subjects. RT-PCR analysis showed decreased gene expression of PPAR-γ2 in morbidly obese individuals (*p* < 0.05). Our present analysis confirms the results other published studies [36] that PPAR-γ2 expression was decreased in VAT [29]. This profile was insulin-dependent in morbidly obese patients, since HIR-MO individuals showed decreased expression levels in comparison with LIR-MO subjects (Figure 2a) (*p* < 0.05). Consistently with the RT-PCR analysis, the immunohistochemical analysis showed similar results according to the PPAR-γ2 expression profile. While non-obese individuals showed strong staining and more positive immunoreactivity, the morbidly obese individuals (LIR-MO + HIR-MO) had a moderate/weak staining and less immunoreactivity for PPAR-γ2 (Figure 2b).

To amplify the findings of the immunohistochemical analysis and verify the recruitment of PPAR-γ2 and RXR-α as a heterodimer to activate specific adipocyte gene expression, we next investigated its activation in VAT in MO to determine its association with insulin resistance. For this purpose, we performed a series of co-immunoprecipitation experiments using nuclear extracts from VAT from morbidly obese individuals, treated with rosiglitazone before co-immunoprecipitation. This assay showed a positive staining for PPAR-γ2 that was co-immunoprecipitated with RXR-α in VAT from morbidly obese individuals. However, the LIR-MO subjects showed strong staining in comparison with HIR-MO individuals (Figure 2c, Lanes 3 and 6). Furthermore, these results were similar to the mRNA expression and immunohistochemistry of PPAR-γ2 in HIR-MO and LIR-MO subjects.

### 3.4. PPAR-γ2 Activity is Reduced in VAT from Morbidly Obese Individuals and Associated with Insulin Resistance

**Figure 3:**
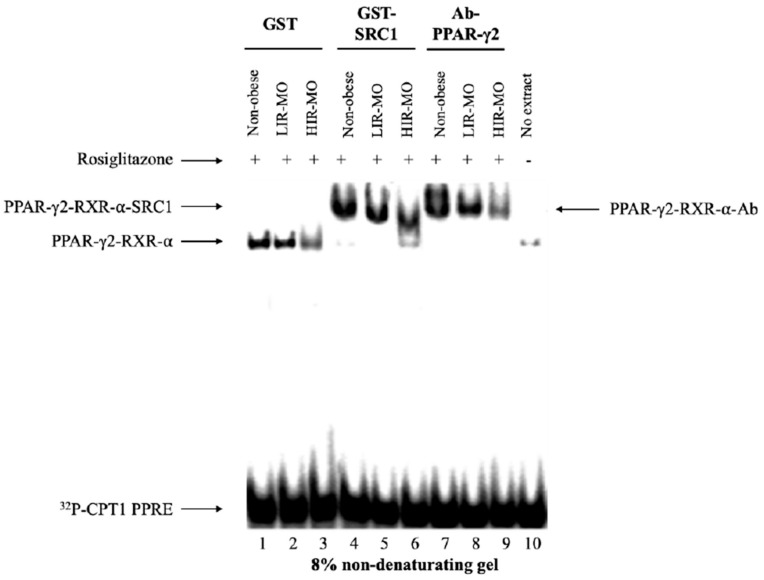
PPAR-γ2 activity in VAT: A specific-pathway view. Combined gel shift and supershift experiments were performed with nuclear extracts from VAT from non-obese (Lanes 1, 4 and 7) (*n* = 15), LIR-MO (Lanes 2, 5 and 8) (*n* = 11) and HIR-MO (Lanes 3, 6 and 9) (*n* = 12) individuals, to test the binding ability of PPAR-γ2–RXR-⍺ heterodimer to the ^32^P-labeled CPT1 PPRE probe. Nuclear extracts were pre-incubated with rosiglitazone. After that, we added 5–10 μg of bacterially expressed wild-type GST (Lanes 1–3), GST-SRC1 (Lanes 4–6), and specific anti-PPAR-γ2 antibodies (Lanes 7–9). Protein–DNA complexes were revealed through 8% non-denaturing polyacrylamide gels. Lane 10 corresponds the no extract-free probe, used as a negative control in this assay. Abbreviations: VAT, Visceral adipose tissue; PPAR-γ2, peroxisome proliferator-activated receptor γ type 2; LIR-MO, Low insulin resistance-morbid obese; HIR-MO, High insulin resistance-morbid obese; RXR-⍺, Retinoid X receptor type α; GST, Glutathione S-transferase; GST-SRC1, Glutathione S-transferase steroid receptor co-activator type 1.

To test PPAR-γ2 activity through RXR-α interaction, we first compared whether PPAR-γ2 could bind as a heterodimer with RXR-α and activate the functional PPRE sites on DNA by EMSA assay. The last lane represents the probe alone without nuclear extract, used as a negative control (Figure 3, Lane 10). The binding affinity of the PPAR-γ2–RXR-α heterodimer to the functional PPRE site was decreased in HIR-MO individuals (Figure 3, Lane 3), in comparison with LIR-MO (Figure 3, Lane 2) and non-obese subjects (Figure 3, Lane 1). In fact, this affinity of PPAR-γ2–RXR-α heterodimer to recruit the co-activator GST-SRC1 was remained markedly decreased in HIR-MO subjects (Figure 3, Lane 6). Since many transcription factors can bind to DR-1 type PPRE sites, and that could induce errors when interpreting the data, we next performed supershift assay with specific anti-PPAR-γ2 antibodies to test the specificity of our results. As shown in Figure 3, the amount of supershifted complex (PPAR-γ2–RXR-α) on CPT1 gene on DR1-type PPRE site was also decreased in HIR-MO individuals (Figure 3, Lane 9).

### 3.5. PPAR-γ2–RXR-α Physically Interact with the IGFBP-2 Promoter through the Functional PPRE Domain

**Figure 4:**
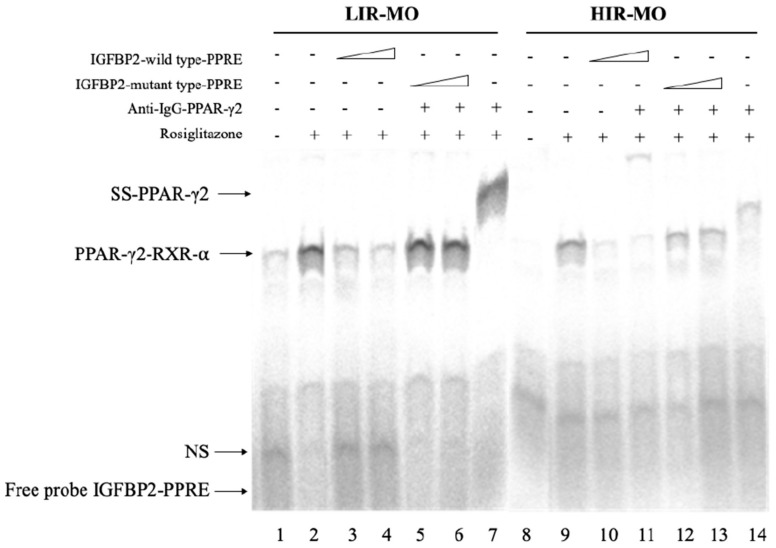
IGFBP-2 and PPAR-γ2: A tight collaboration in insulin resistance pathway. Specific binding activity of PPAR-γ2 to the putative IGFBP2–PPRE sequence was shown using EMSA assay by non-radioactive method. Nuclear extract from VAT from LIR-MO (*n* = 11) and HIR-MO (*n* = 12) individuals was incubated in the presence of rosiglitazone. We used both wild-type non-labeled IGFBP2–wild type-PPRE and mutant non-labeled IGFBP2–mutant type-PPRE. Combined gel shift and supershift experiments were performed. Lane 1 of the panel represents the probe alone without nuclear extract, used as a negative control of the system. Abbreviations: PPAR-γ2, activated receptor γ type 2; VAT, Visceral adipose tissue; LIR-MO, Low insulin resistance-morbid obese; HIR-MO, High insulin resistance-morbid obese. SS- PPAR-γ2, Supershift PPAR-γ2.

We tested by EMSA whether PPAR-γ2 was able to bind to IGFBP-2 promoter sequence in an insulin-dependent manner, in VAT from morbidly obese individuals. For all that, double-stranded IGFBP-2–wild type-PPRE and IGFBP-2–mutant type-PPRE oligonucleotides were incubated with nuclear extracts of VAT from LIR-MO and HIR-MO individuals. Our results show that a weak specific DNA–PPAR-γ2–RXR-α complex was formed in the presence of IGFBP2–PPRE, in HIR-MO (Figure 4, Lane 9) in comparison with LIR-MO individuals (Figure 4, Lane 2). To confirm the specific presence of PPAR-γ2 in the IGFBP2–PPRE complex, anti-PPAR-γ2 antibodies were added for supershift assays. We observed that the IGFBP2–PPRE sequence was bound by proteins recognized by PPAR-γ2 antibodies, thus producing a supershift complex, weaker in HIR-MO (Figure 4, Lane 14) than LIR-MO individuals (Figure 4, Lane 7). In addition, when a 500-fold molar excess of unlabeled oligonucleotide of IGFBP2–wild type-PPRE, was added, the signal was diminished (Figure 4, Lanes 3 and 4 for LIR-MO subjects and Lanes 9 and 10 for HIR-MO subjects). In contrast, when an unlabeled oligonucleotide containing mutations in the 5′sequence (IGFBP2–mutant type-PPRE) was added, the DNA-binding was no longer competed (Figure 4, Lanes 5 and 6 for LIR-MO subjects and Lanes 12 and 13 for HIR-MO individuals). No complex was formed when nuclear extract was not added (Figure 4, Lane 1 for LIR-MO individuals and Lane 9 for HIR-MO subjects).

## 4. Discussion

Most published gene expression analyses to determine a specific expression profile for obesity were done in typical metabolic tissues such as adipose tissues, muscle or liver. However, VAT, as an interesting metabolic tissue to notice that some of the differentially regulated genes, have strong associations not only with obesity, but with important comorbidities, such as insulin resistance. In our study, we have demonstrated that the expression of C/EBP-α is decreased in VAT from morbidly obese subjects. This expression profile seems to have a close association with insulin sensitivity, since HIR-MO individuals showed decreased expression patterns of C/EBP-α when compared to with LIR-MO subjects. Indeed, a study reported that the expression of C/EBP-α in human adipose tissue was correlated with metabolic parameters, suggesting a tightly cross-regulation of C/EBP-α in adipogenesis and insulin sensitivity [37].

The physiological/clinical role of C/EBP-α has previously been extensively studied during adipose tissue differentiation. Although both insulin and C/EBPα have been known to regulate gluconeogenesis, the link between insulin and C/EBPα has not been clarified yet [13]. However, the role of C/EBP-α in the liver is well established, since it is involved in many metabolic processes such as glucose and lipid metabolism [38]. Nevertheless, our findings support the relevance of C/EBP-α could be a potential aspirant to protect against insulin resistance in an alternative pathway associated with adipocyte differentiation, and maybe its participation could partially be, one of the possible links between adipogenesis and insulin resistance. As the C/EBPα expression alone cannot explain the expression of metabolic and specific adipogenic-genes in VAT, involved in adipogenesis and insulin resistance, additional evidence is necessary for the dramatic dysregulation of metabolic genes [7]. For that, we next added that FOXO1 expression in VAT was significantly overexpressed in morbidly obese individuals. In fact, FOXO1 expression was greater in HIR-MO individuals in comparison with LIR-MO subjects, suggesting a possible relationship between FOXO1 and insulin resistance during adipocyte differentiation. Indeed, a recent study has shown that FOXO1 links insulin signaling to C/EBP-α and regulates gluconeogenesis, by directly interacting, which FOXO1 bind to the response elements of C/EBP-α, only in the presence of C/EBP-α. Furthermore, the C/EBP-α–FOXO1 complex, may exhibit different functions, depending on the cellular context [18].

FOXO1 actively participates in the development of insulin resistance by modulating the transcription of several transcription factors involved in adipogenesis and lipid and glucose metabolism. This modulation results in an increasing rate of gluconeogenesis and production of glucose by the liver, resulting in an increasing of glucose and insulin production [39,40]. Alternatively, previous studies have reported that FOXO1 represses PPAR-γ transactivation in an insulin-dependent pathway [41]. This repression is carried out by directly FOXO1–PPAR-γ interactions. FOXO1 antagonizes PPAR-γ activity and vice versa, indicating a reciprocal antagonistic manner between FOXO1 and PPAR-γ, by disrupting of DNA binding sites of the heterodimer PPAR-γ–RXR-α [42]. In this study, we found that PPAR-γ2 was decreased in VAT from morbidly obese individuals. Within the MO group, the HIR-MO subjects showed decreased levels of PPAR-γ2 both free protein and as PPAR-γ2–RXR-α heterodimer, suggesting that PPAR-γ2 are also associated with insulin resistance during adipocyte development, and confirming that PPAR-γ2, similar to C/EBP-α and FOXO1, could be a potential candidate to protect against insulin resistance pathways during adipocyte differentiation. 

In fact, PPAR-γ activation through agonists PPAR-γ markedly improves whole-body insulin sensitivity, leading to improved circulating insulin and glucose levels. The mechanism by which PPAR-γ improves insulin sensitivity is complex, because it involves many tissue types, such as adipose tissue, skeletal muscle or the liver. It is known that PPAR-γ activation affects the insulin pathway through direct modulation of specific gene expression such as adipocyte fatty acid binding protein (aP2), phosphoenolpyruvate carboxykinase (PEPCK), lipoprotein lipase (LPL) or fatty acid transport protein (FATP) [43]. These genes increase free fatty acid uptake, clearance and recycling, since is one of the mechanisms through which PPAR-γ can improve the systemic insulin sensitivity. PPAR-γ are also able to activate the AMPK pathway. The activation of AMPK decreases the level of plasma glucose and triglycerides, leading to increase the expression of genes involved in fat oxidation, resulting in the improvement of the systemic insulin sensitivity as well [44]. PPAR-γ2 also cooperates with C/EBP-α, to activate gene expression involved in adipogenesis and insulin sensitivity, since C/EBP-binding sites are found in the promoters of PEPCK, aP2 and GLUT4 [8]. Thus, there must be a tight cross-regulation C/EBP-α, FOXO1 and PPAR-γ2 that modulates adipogenesis linked to insulin resistance in an alternative insulin pathway and common alternative pathway, since it has been reported that they physically interact, cooperate and activate each other. 

As PPAR-γ binds as heterodimers with RXR-α to PPREs [45], we further investigated the activation of the PPAR-γ2–RXR-α heterodimer in the promoter region of the CPT1 gene in the context of obesity related to insulin resistance. Our results show that the formation of the PPAR-γ2–RXR-α complex was weakly staining in HIR-MO individuals, according to the gene expression analysis, co-immunoprecipitation, immunohistochemistry and EMSA assay. Our study suggests that the activity of the PPAR-γ2–RXR-α heterodimer, either through the increased activation of co-activators, specific co-activator or specific PPAR-γ agonists, could have an interesting role in obesity and obesity related-insulin resistance. We also found for the first time that PPAR-γ–RXR-α heterodimer was able to bind to the IGFBP-2 promoter to modulate its transcriptional activity. In fact, this activation was dependent to insulin resistance grade, since the HIR-MO individuals showed less staining than LIR-MO subjects, suggesting that HIR-MO subjects have less activity of PPAR-γ–RXR-α, and further, less activity of IGFBP-2. Our experiments showed that PPAR-γ2 only interacts with IGFBP-2, of the IGFBP family (IGFBP-1–4, data not shown).

Indeed, several studies have reported that circulating IGFBP-2 was related to insulin sensitivity, metabolic syndrome and antidiabetic effect by regulate the expression of leptin gene [46,47,48]. For instance, the activation of PPAR-γ2, which cannot be blocked by FOXO1, may up-regulated IGFBP-2 expression to protect adipocyte cells towards metabolic alterations associated with obesity. Although the IGFBP-2 gene transcription is regulated by several metabolic conditions in endocrine organs, the underlying regulatory mechanism of IGFBP-2 gene expression remains unknown, and further studies are needed [49]. Moreover, the functional roles of IGFBP-2 in metabolic diseases and signaling are still unclear and controversial.

A study has showed the capacity C/EBP-α to activate the expression of IGFBP-2. In this way, insulin stimulates IGFBP-2 mRNA levels in mature 3T3-L1 adipocytes through the stimulation of PI3K and mTOR, which induces C/EBP-α recruitment and transactivation of the IGFBP-2 promoter [24]. Indeed, IGFBP-2 promoter already has a binding site for C/EBP-α, which indicates that they physically interact. A study conducted by Heald et al. indicated that IGFBP-2 circulating levels are a solid marker of metabolic syndrome in humans in individuals with type 2 diabetes. Blood IGFBP-2 levels was also correlated negatively with some lipid markers, such as triglycerides and total cholesterol, and positively with insulin sensitivity. Thus, IGFBP-2 might protect against the development of obesity and insulin resistance in humans. Therefore, the relevance of IGFBP-2 to metabolic disorder could be a main driver in the investigation of IGFBP-2’s underlying physiological function and may be an important candidate to protect against insulin resistance to diabetes, in obese individuals. However, more studies are needed to clarify the physiological/clinical role of this association.

Our study had several limitations, among which was the small sample size. Although the number of study participants met the requirement for analysis, the present sample size was relatively small. However, it would be interesting to provide more body composition characteristics of the participants and put in context the results presented in this study. Moreover, data from subcutaneous adipose tissue and culture cells may provide other insights about the expression patterns of these genes, especially the effect of IGFBP-2 and its activation by PPAR-γ. Because no data about the interaction of IGFBP-2 and PPAR-γ have been reported, the evidence cannot rule out the importance of PPAR-γ2–IGFBP-2 interaction, especially in insulin resistance and obesity. Thus, more specific studies in obese individuals with insulin resistance will be necessary to confirm these findings using transcriptomic tools to put in context the physiological/clinical role of PPAR-γ2–IGFBP-2 interaction. 

We have presented in this study that C/EBP-α, FOXO1, PPAR-γ2 and IGFBP-2 have an interesting association in insulin resistance associated with obesity: their expression profile was found altered in adipose tissue in an insulin-dependent manner. Thus, these genes might provide a novel characteristic to protect against the development of obesity and improve insulin sensitivity during adipogenesis.

## 5. Conclusions

We provide data that support the idea that C/EBP-α, FOXO1, PPAR-γ2 and IGFBP-2 in visceral adipose tissue are related to obesity-related insulin resistance. We show a down-expression of C/EBP-α and PPAR-γ2 and an overexpression of FOXO1 in VAT from high insulin resistance morbidly-obese (HIR-MO), and weak binding of PPAR-γ2 to RXR-α to form heterodimer in HIR-MO. Finally, PPAR-γ2–RXR-α heterodimer was able to bind to IGFBP-2 promoter, but it was weaker in HIR-MO subjects. Our study suggests the relevance of better understanding these transcription factors in visceral adipose tissue to determine their possible role in the etiology of obesity-related insulin resistance in an insulin-independent manner, which could lead to new therapeutic and anti-diabetic strategies.

## Figures and Tables

**Table 1:** Anthropometric and biochemical variables of non-obese and morbidly obese patients.

Variables	Non-obese	LIR-MO	HIR-MO
**Sex (male/female)**	15 (8/7)	11 (5/6)	12 (5/7)
**Age (years)**	47.0 ± 15.6	45.6 ± 11.7	37.8 ± 9.6
**Weight (kg)**	66.2 ± 11.8 ^a,b^	150.0 ± 26.9	156.2 ± 18.7
**Waist circumference (cm)**	83.3 ± 10.5 ^a,b^	141.0 ± 16.3	143.7 ± 20.2
**BMI (kg/m^2^)**	23.1 ± 2.45 ^a,b^	56.5 ± 7.1	55.4 ± 3.9
**Insulin (µIU/mL)**	9.2 ± 3.9 ^a,b^	14.1 ± 4.0 ^c^	44.5 ± 7.8
**Glucose (mg/dL)**	84.6 ± 14.7 ^b^	93.0 ± 10.1 ^c^	102.3 ± 10.9
**HOMA-IR**	1.99 ± 0.09 ^a,b^	3.27 ± 0.94 ^c^	11.28 ± 2.43
**Cholesterol (mg/dL)**	193.2 ± 44.0	204.5 ± 39.8	200.4 ± 23.5
**Triglycerides (mg/dL)**	90.3 ± 50.4	111.3 ± 35.2	136.4 ± 91.2
**HDL-cholesterol (mg/dL)**	55.5 ± 17.2	46.8 ± 14.8	42.2 ± 17.9
**LDL-cholesterol (mg/dL)**	119.6 ± 37.2	135.4 ± 29.9	130,9 ± 44.2

The results are given as the mean ± SD. Different letters indicate significant differences between the means of the different groups of subjects (*p* < 0.05; a, non-obese vs. LIR-MO; b, non-obese vs. HIR-MO; c, LIR-MO vs. HIR-MO) according to Student’s *t*-test. Abbreviations: LIR-MO, Low insulin resistance-morbidly obese individuals; HIR-MO, High insulin resistance-morbidly obese subjects; BMI, Body mass index; HDL, High density lipoprotein; LDL, low density lipoprotein; HOMA-IR, Homeostasis model assessment of insulin Resistance.
